# Embryonic Temperature Programs Phenotype in Reptiles

**DOI:** 10.3389/fphys.2020.00035

**Published:** 2020-01-31

**Authors:** Sunil Kumar Singh, Debojyoti Das, Turk Rhen

**Affiliations:** Department of Biology, University of North Dakota, Grand Forks, ND, United States

**Keywords:** endocrinology, growth, sex determination, temperature, thermal acclimation

## Abstract

Reptiles are critically affected by temperature throughout their lifespan, but especially so during early development. Temperature-induced changes in phenotype are a specific example of a broader phenomenon called phenotypic plasticity in which a single individual is able to develop different phenotypes when exposed to different environments. With climate change occurring at an unprecedented rate, it is important to study temperature effects on reptiles. For example, the potential impact of global warming is especially pronounced in species with temperature-dependent sex determination (TSD) because temperature has a direct effect on a key phenotypic (sex) and demographic (population sex ratios) trait. Reptiles with TSD also serve as models for studying temperature effects on the development of other traits that display continuous variation. Temperature directly influences metabolic and developmental rate of embryos and can have permanent effects on phenotype that last beyond the embryonic period. For instance, incubation temperature programs post-hatching hormone production and growth physiology, which can profoundly influence fitness. Here, we review current knowledge of temperature effects on phenotypic and developmental plasticity in reptiles. First, we examine the direct effect of temperature on biophysical processes, the concept of thermal performance curves, and the process of thermal acclimation. After discussing these reversible temperature effects, we focus the bulk of the review on developmental programming of phenotype by temperature during embryogenesis (i.e., permanent developmental effects). We focus on oviparous species because eggs are especially susceptible to changes in ambient temperature. We then discuss recent work probing the role of epigenetic mechanisms in mediating temperature effects on phenotype. Based on phenotypic effects of temperature, we return to the potential impact of global warming on reptiles. Finally, we highlight key areas for future research, including the identification of temperature sensors and assessment of genetic variation for thermosensitivity.

## Introduction

All species have adapted, in one way or another, to natural variation in their environment. Variation in photoperiod, temperature, precipitation, salinity of soil or water, oxygen concentration, fires, and other organisms like pathogens, predators, and prey occurs across a wide range of spatial and temporal scales. Life on Earth has also faced major challenges during its history, including five mass extinctions caused by natural, albeit rare, events like extreme volcanic activity and the impact of very large meteors (Barnosky et al., 2011). Over the last century, humans have had a dramatic impact on the environment, causing rapid changes in climate, destroying habitat, and producing entirely new challenges that may be causing a sixth mass extinction (Barnosky et al., 2011; Scheffers et al., 2016; Waters et al., 2016). For example, human generated pollution and novel synthetic chemicals that act as endocrine disruptors are major causes of disease and death both in people and in wildlife (Gore et al., 2015; Landrigan et al., 2018). We need to understand, now more than ever, how animals and plants respond to natural and anthropogenic variation in the environment in order to predict, prevent, and/or mitigate negative impacts on our biosphere.

On one hand, organisms can maintain phenotypic stability when exposed to complex and variable environments. Physiologists refer to this as homeostasis while developmental biologists call it canalization. On the other hand, organisms may display different phenotypes in the face of environmental challenges, a phenomenon called phenotypic plasticity (Pfennig et al., 2010). Although plasticity and stability appear to be distinct strategies for dealing with environmental variation, they actually represent two ends of a continuum of potential responses. Indeed, plasticity/stability has a genetic basis with different individuals being more or less responsive to environmental influences (Via and Lande, 1985; Scheiner, 1993). Think, for instance, of people that smoke tobacco and never develop lung cancer, and others that develop lung cancer from exposure to lower levels of second-hand smoke (Warkentin et al., 2017). Such individuals have very different responses to their environment. A clear understanding of the genetic and molecular mechanisms that organisms use to maintain a stable phenotype or to change their phenotype has broad implications in areas as diverse as medicine and climate change. Here we focus on phenotypic responses to temperature, a key environmental variable that affects every living organism.

**Figure 1:**
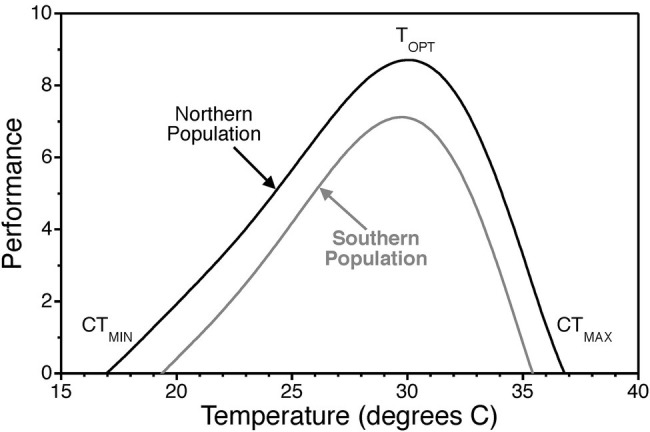
Thermal performance curve illustrating the typical pattern of physiological performance as a function of temperature. Thermal performance curves are characterized by an optimal temperature T_OPT_ where performance is maximized. They also exhibit critical thermal minima CT_MIN_ and critical thermal maxima CT_MAX_ where performance declines to zero. Scales on the x and y axes are arbitrary and simply used to illustrate general patterns of thermal responsiveness. Optimal temperatures, the range from critical thermal minima to the critical thermal maxima, and the width of curves vary across biological scales of organization, among individuals, among populations (i.e., countergradient variation), and among species.

Temperature, *via* its direct effect on the rate of biochemical reactions, influences biological processes from the cellular to the organismal level (Gillooly et al., 2002; Kingsolver, 2009; Schulte et al., 2011). Within the range of body temperatures of most organisms (0–40°C), biochemical reaction rates increase with increasing temperature until a point where stability of biological molecules is compromised and reaction rates drop. This general pattern translates to higher levels of biological organization and is referred to as a thermal performance curve (Figure 1). In turn, temperature effects on individual organisms drive ecological processes (Stenseth et al., 2002; Brown et al., 2004).

The Intergovernmental Panel on Climate Change (IPCC, 2013) lays out unambiguous physical and biogeochemical evidence that global temperatures have been increasing since the late 1900s. Direct measurements demonstrate that land surface air temperatures, sea surface air temperatures, troposphere temperature, and ocean heat content have been increasing rapidly since 1980. During the same period, glacier mass balance and the extent of summer arctic sea ice have been declining. Even as global averages are increasing, there is spatial variation with different regions being more or less severely affected by climate change (Bellard et al., 2012).

Climate change and heterogeneity in temperature are also observed at finer spatial and temporal scales. For example, Maclean et al. (2019) estimated and analyzed hourly air temperatures at a 100-m resolution on the Lizard Peninsula, United Kingdom over nearly four decades. While there was clear warming of climate across the entire 17 square kilometer area, they found lower rates of warming on north-east facing slopes, substantial spatial variation in the number of frost-free days, and five-fold differences in the number of growing degree-days. Diurnal and seasonal patterns of temperature change normally serve as cues and as drivers of major life history events in both plants and animals (Peñuelas and Filella, 2001; Badeck et al., 2004). Body temperature, for instance, strongly influences growth rate in ectotherms (Angilletta et al., 2002). Growth rate, in turn, influences age and size at reproductive maturity (Stearns, 1992). Increasing mean temperatures and shifting patterns of thermal variance in space and time have already had a demonstrable effect on terrestrial and marine ecosystems (Parmesan and Yohe, 2003; Burrows et al., 2011).

Temperature effects on organismal phenotype are especially clear in taxonomic groups like reptiles that do not produce their own heat and have variable body temperatures (i.e., ectothermic poikilotherms). Reptiles live in a wide range of climates from tropical to sub-tropical to northern temperate zones and are susceptible to temperature effects throughout their lifespan. Although juvenile and adult reptiles can thermoregulate *via* basking and selecting thermally distinct microhabitats and through physiological mechanisms that influence heat transfer (Adolph, 1990; Seebacher and Franklin, 2005), variation in body temperature is still substantial. Reptiles are also able to respond to temperature through the process of acclimation, which involves longer-term physiological changes that compensate for direct temperature effects on reaction rates (e.g., increases in enzyme expression to maintain the same overall rate of metabolism at a lower temperature). Acclimation is a reversible response that occurs over days or weeks in association with seasonal changes in temperature. Temperature can also have permanent developmental effects on phenotype, as observed in species with temperature-dependent sex determination. Finally, changes in thermal environment can exert selection pressure on thermal performance curves and cause evolutionary adaptation (Angilletta and Angilletta, 2009; Logan et al., 2014).

Countergradient variation in growth rate, for instance, provides strong evidence of genetic adaptation to geographic gradients in environmental variables (Conover and Schultz, 1995). In brief, countergradient variation refers to genetic differences among populations that counteract the phenotypic effects of the environmental gradient. In the case of thermal gradients with latitude in the Atlantic silverside, northern populations in cooler environments exhibit faster growth than southern populations in warmer environments when tested at the same temperatures (Conover and Present, 1990). This type of genetic adaptation would manifest as an upward shift in the thermal performance curve for growth rate such that more northern populations perform at the same level even though they live in cooler environments (Figure 1). It is important to note that countergradient variation is due to genetic differentiation and is not a result of physiological acclimation.

Here we briefly review the physics of temperature effects on biochemical reactions, the general pattern of thermal effects on physiology, and the process of thermal acclimation in ectotherms. However, our main focus is developmental programming of phenotype by temperature during embryogenesis (i.e., permanent developmental effects). We concentrate our review on studies of egg laying species because embryos in eggs are particularly susceptible to changes in ambient temperature and have minimal ability to thermoregulate (Telemeco et al., 2016; Cordero et al., 2018). Variation in incubation temperature in natural nests depends on the ecological conditions around the nesting area, location of the nest (shaded or open), the depth of eggs in the nest, and nesting phenology (Weisrock and Janzen, 1999; Shine and Elphick, 2001; Booth, 2006). Reptile eggs serve as an ideal model to study thermal effects because temperature can be easily manipulated. In contrast, females in live bearing lizards like *Zootoca vivipara* can behaviorally thermoregulate and alter the temperature their offspring experience (Foucart et al., 2018). Some egg-laying reptiles like prairie skinks and water pythons even brood their eggs to affect incubation temperature, which further highlights the importance of developmental temperature during embryogenesis (Lang, 1990; Shine et al., 1997).

## Biophysical Effects of Temperature

Temperature effects on biological systems can be ephemeral or they can be long-lasting. Short-lived effects are mediated by the direct, instantaneous, and reversible effect of temperature on the rate of biochemical reactions. Reaction rates increase with increasing temperatures, up until the point that elevated temperatures begin to disrupt the structure of the proteins (or other biomolecules) involved in the biological process. In addition to ephemeral effects, temperature can have lasting effects that persist well after ambient temperature changes. Persistent temperature effects on phenotype are mediated by post-translational modifications to proteins and/or changes in gene expression. These temperature-induced changes may be reversible over longer timescales (i.e., physiological acclimation) or they can be permanent and last a lifetime (i.e., developmental plasticity or polyphenism). It is therefore important to distinguish between different timescales to fully understand thermal biology (Schulte et al., 2011).

We begin our discussion of temperature at its most fundamental biophysical level. In order to quantitatively study the sensitivity of living organisms to temperature changes, it is customary to use the temperature coefficient (Q_10_) value (Hegarty, 1973), which is a measure of the rate of change of a biological or chemical system due to temperature increase of 10°C.

$${\text{Q}}_{10}={\left({\text{R}}_{2}/{\text{R}}_{1}\right)}^{10\text{ degrees C}/\left(\text{T}2-\text{T}1\right)}$$

where R_1_ is the rate of the reaction at temperature T_1_, R_2_ is the rate of the reaction at temperature T_2_, and temperature is measured in degrees Celsius. A Q_10_ value of 1 indicates thermal independence, values above 1 indicate that reaction rates increase with increasing temperature, while values below 1 indicate that reaction rates decrease with increasing temperature. Q_10_ values for biological systems are typically between 2 ~ 3 when examined in the normal range of body temperatures for a species (i.e., the part of thermal performance curves between the critical thermal minimum and the optimal temperature). There have been few studies of metabolic rate in reptile embryos, but they also report Q_10_’s around 2 (Angilletta, 2001; Angilletta et al., 2006).

To understand the rationale behind Q_10_ values, one needs to digress to the collision rate theory. According to this theory, the rate constants for chemical reactions are a direct function of temperature. Reactions are, at an elementary level, due to collisions of reactants with a certain threshold energy. Now, with an increase in thermal energy, the kinetic energies of the reactants increase resulting in more frequent collisions and an increase in the reaction rate. However, biochemical reactions and interactions involve proteins and other biomolecules (e.g., functional RNAs) whose three-dimensional shape depends unequivocally on non-covalent intra-molecular interactions. The stability of biomolecules is diminished at elevated operational temperatures. As these molecules start to denature at higher temperatures, reaction rates drop. This results in thermal performance curves with an optimal temperature at which reaction rate is maximized (Figure 1). Critical thermal minima and maxima for thermal performance curves are defined as the temperatures where reaction rates drop to zero (Figure 1). Thus, Q_10_ values for biochemical processes are not a monotonic function of temperature. Several mathematical models have been developed that incorporate both the temperature dependence of reaction kinetics described by the collision rate theory (i.e., the Arrhenius equation) as well as temperature effects on enzyme stability (DeLong et al., 2017).

While this general pattern of thermal performance extends beyond simple biochemical reactions to higher levels of biological organization, the precise pattern of acute temperature effects varies because cells, tissues, organs, and organisms are complex, hierarchical systems. Performance curves at higher levels of organization vary in part because different subcomponents (e.g., biomolecules within cells) can display different thermal performance curves and the combined effect of subcomponents may be nonlinear and/or non-additive with regard to performance of the system as a whole. We refer readers to Gangloff and Telemeco (2018) for an in-depth review of thermal performance curves at higher levels of biological organization. These authors also propose a novel hypothesis, Hierarchical Mechanisms of Thermal Limitation, that integrates thermal effects on subcellular components and organ systems to better understand thermal tolerance at the whole organism level. Moreover, cells, tissues, organs, and whole organisms are dynamic systems that can sense and respond to their thermal environment through physiological acclimation (Somero, 2005; Keen et al., 2017; Rohr et al., 2018).

## Thermal Acclimation in Juveniles and Adults

Acclimation occurs when prolonged exposure to a new thermal regime, be it in nature or in a controlled laboratory setting, induces alterations in the acute response to temperature (i.e., the thermal performance curve changes). As mentioned previously, exposure to cold temperatures can induce physiological changes that compensate for the direct negative effect of low temperature on reaction rates. A well-studied example is remodeling of the heart in fish species that remain active during the cold season. Cold-induced changes in gene expression alter structural and functional properties of the heart, from the sub-cellular level (e.g., myofilaments, electrical activity, and calcium handling) to the cellular level (e.g., myocyte contraction) to the organ level (e.g., gross changes in heart size and stiffness) (reviewed by Keen et al., 2017). Together, these changes preserve whole organism performance across a broader range of temperatures than would otherwise be possible. Similar effects of cold acclimation on the cardiovascular system are observed in turtles (Risher and Claussen, 1987; Keen et al., 2016). Heat acclimation is also a well-established phenomenon in which exposure to elevated temperatures increases the critical thermal maximum of a broad range of organisms (Sinclair et al., 2016; Rohr et al., 2018), including lizards, snakes, and turtles (Murrish and Vance, 1968; Jacobson and Whitford, 1970; Williamson et al., 1989; Brown, 1996). In short, acclimation entails shift in thermal optima, critical thermal minima and maxima, and/or changes in the breadth of thermal performance curves (Rohr et al., 2018). Thermal acclimation in ectotherms is a form of phenotypic plasticity that reduces performance differences in thermally variable environments.

## Developmental Effects of Temperature

**Figure 2:**
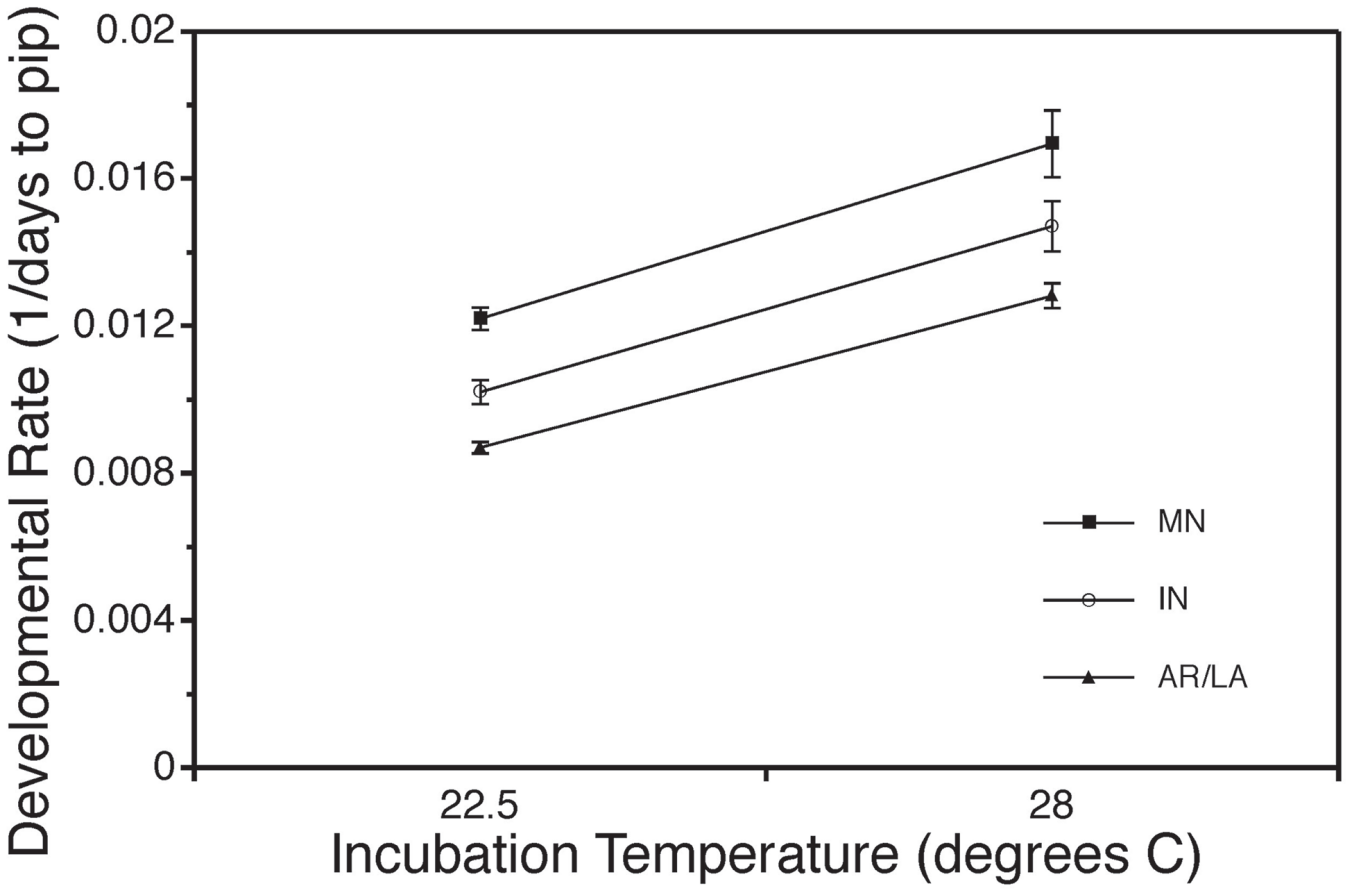
Countergradient variation for developmental rate of embryonic common snapping turtles, *Chelydra serpentina*. Eggs from Minnesota, Indiana, and Arkansas/Louisiana were incubated at the same temperatures, 22.5 or 28°C. Incubation period was recorded as the number of days from oviposition to pipping. Developmental rate is the inverse of incubation period (1/days to pip). Data are modified from Ewert et al., 2005.

Temperature also has complex effects on the development of reptilian embryos. On one hand, developmental rate displays a classic thermal response. As incubation temperature increases, reptile embryos develop faster and hatch sooner (reviewed in Noble et al., 2018). This pattern is clearly seen in snapping turtle embryos, which also display evidence of countergradient variation in developmental rate (Figure 2; Ewert et al., 2005). Yet, hatchlings incubated at different temperatures are not simply exact replicas of each other (Rhen and Lang, 2004; Booth, 2018; Mitchell et al., 2018; Noble et al., 2018; While et al., 2018). Animals from different incubation temperatures are marked by differences in body size, body shape, amount of residual yolk, size of fat stores, locomotor performance, thermoregulatory behavior, sexual phenotype, and many other traits. In turn, incubation temperature effects on traits like critical thermal minima and maxima may have a significant impact on susceptibility of reptiles to global warming, as suggested for the velvet gecko (Dayananda et al., 2017). To understand how these differences arise, we need to examine embryogenesis in more detail.

Embryonic development can be broken down into two basic processes. First is differentiation, wherein specific cell types, tissues, organs, and organ systems are formed. Second is growth, characterized by an increase in size of the embryo due to cell proliferation, cell hypertrophy (for certain cell types), and deposition of extracellular matrix. Another facet of embryogenesis is the simultaneous development of extraembryonic membranes that play a critical role in gas, nutrient, and waste exchange. Development in vertebrates can be further classified in terms of temporal progression of cleavage, gastrulation, neurulation, and organogenesis as well as early growth and late growth.

Given these fundamental processes, temperature most likely has its long-term effects on reptiles by influencing differentiation, growth, and/or the temporal relationships among these processes. In this context, studies in common snapping turtles and the skink *Bassiana duperreyi* have found that early to middle stages of embryogenesis are more susceptible to temperature effects than later stages of development (Yntema, 1968; Birchard and Reiber, 1995, 1996; Shine and Elphick, 2001). This suggests temperature has a greater impact on cell, tissue and organ differentiation, which occurs earlier in development, than it does on growth. The finding that the latter half of development, when embryos grow most rapidly, is less responsive to temperature suggests embryos acclimate to temperature. Studies of heart rate show that embryos in one turtle, one snake, and one lizard species, but not a second lizard species, acclimate to temperature (Du et al., 2010). We start our discussion of permanent developmental effects with an unambiguous example of temperature altering cell and organ fate decisions.

## Temperature-Dependent Sex Determination

Two principal mechanisms of sex determination are routinely encountered in reptiles: genotypic sex determination (GSD) and environmental sex termination (ESD). In GSD species, gonadal sex is determined by genetic factors like sex chromosomes that are inherited at fertilization. In contrast, ESD occurs when external signals determine gonadal sex well after fertilization (Bull and Vogt, 1979; Janzen, 1994). Temperature is the only external cue known to affect sex determination in reptiles (Bull and Vogt, 1979; Janzen, 1994). Even more fascinating is the fact that temperature, a continuous variable, is transduced into a binary response, with individuals normally developing either testes or ovaries (but not as intersexes). As a result, the impact of the thermal environment on sex determination is visualized as a reaction norm in which sex ratios of groups of individuals are plotted as a function of incubation temperature.

**Figure 3:**
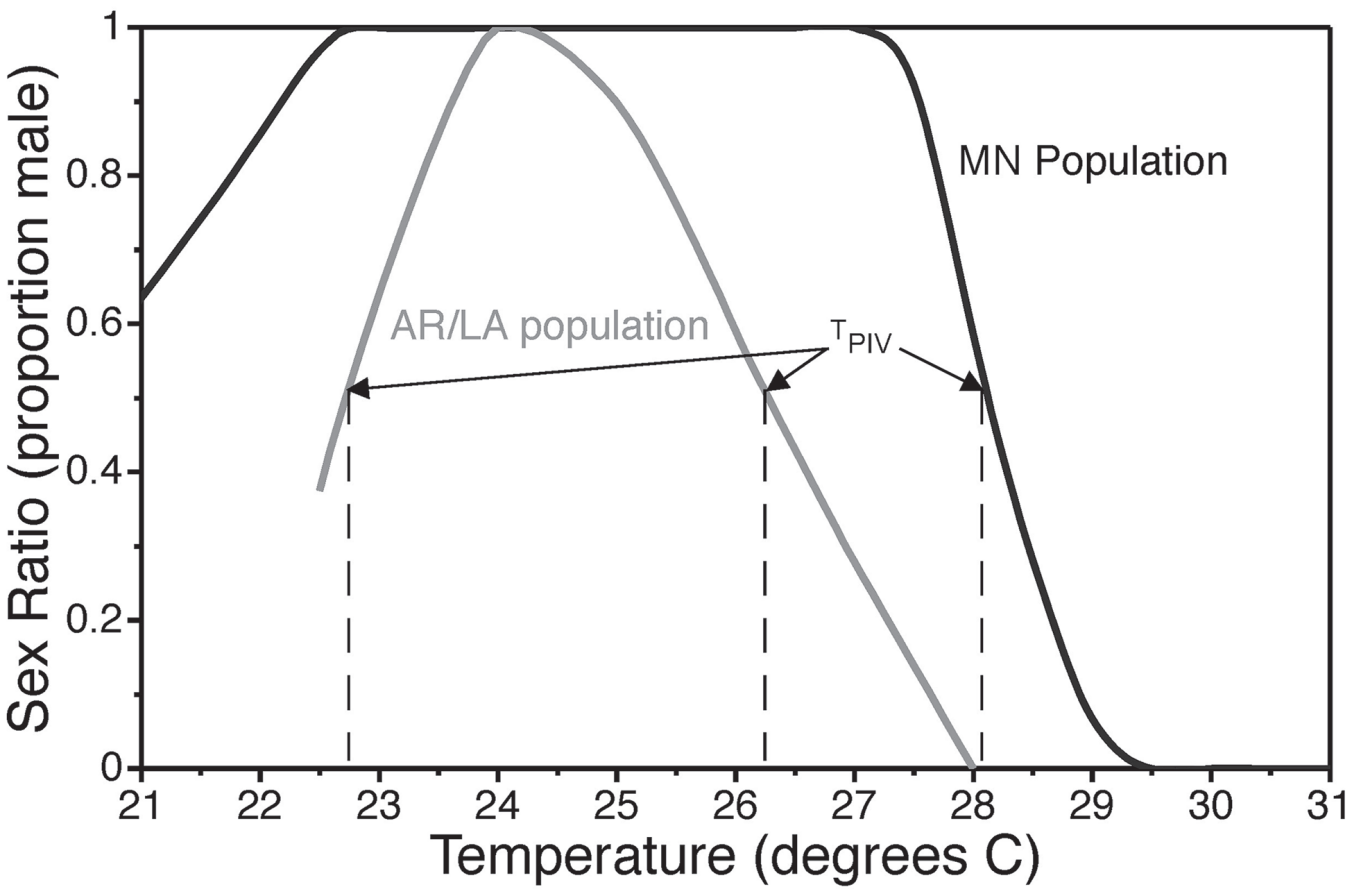
Thermal reaction norm for sex determination in common snapping turtles, *Chelydra serpentina*, from Minnesota and the Arkansas/Louisiana border. The constant incubation temperature that produces a 1:1 sex ratio is called the pivotal temperature T_PIV_. Data are modified from Ewert et al., 2005.

Reaction norms for TSD typically follow one of three fundamental patterns (Ewert and Nelson, 1991). In species with a male-female pattern (MF; a.k.a. pattern Ia), males are produced at low temperatures and females at high temperatures. In species with a female-male pattern (FM; a.k.a. pattern Ib), females are produced at low temperatures and males at high temperatures. The female-male-female pattern (FMF; a.k.a. pattern II) is characterized by female production at low and high temperatures with males developing at intermediate temperatures. Mixed sex ratios are produced within a transitional range between temperatures that induce exclusively one sex or the other. The term pivotal temperature refers to the temperature(s) within the transitional range(s) that produces a 1:1 sex ratio (Mrosovsky and Pieau, 1991). The FMF pattern with two pivotal temperatures has been reported in all groups of reptiles with TSD, viz., turtles, crocodilians, and lizards. Therefore, it is hypothesized that the FMF pattern is ancestral, while the FM and MF pattern are derived patterns resulting from suppression of ovary development and activation of testis development at high or low incubation temperatures (Ewert and Nelson, 1991). The common snapping turtle exhibits a dramatic latitudinal cline in TSD pattern with a gradual change from a clear FMF pattern in southern populations to a MF-like pattern in northern populations (Figure 3; Ewert et al., 2005). The mechanism underlying the evolutionary transition from female to male determination is uncertain because the full gene regulatory network underlying TSD is not known.

Reaction norms for TSD vary both among and within species (reviewed by Deeming, 2004; Ewert et al., 2004; Harlow, 2004; Roush and Rhen, 2018), which suggests that TSD is a threshold trait determined by a combination of genetic and environmental factors. There are differences in pivotal temperature(s) among clutches of eggs produced by different females within populations and among populations from different geographic regions (reviewed in Roush and Rhen, 2018). Variation in pivotal temperatures is correlated with latitude in some species (Ewert et al., 2005; Schroeder et al., 2016), but not others (Bodensteiner et al., 2019). In those species that exhibit geographic clines, pivotal temperatures tend to increase with an increase in latitude (Bull et al., 1982; Vogt and Flores-Villela, 1992; Ewert et al., 2005).

There are two primary hypotheses about the underlying cause of intraspecific variation in pivotal temperatures: the first is that there is genetic variation in temperature sensitivity of embryos and the second is that non-genetic maternal factors cause variation in sex ratio of offspring (e.g., steroid hormones deposited in egg yolk prior to oviposition). To date, few studies have rigorously addressed these hypotheses (reviewed in Roush and Rhen, 2018). We have therefore used controlled breeding studies to tease apart additive genetic effects from genetic variance due to allelic dominance and non-genetic maternal effects. We found that variation in TSD in leopard geckos and common snapping turtles is genetically based and is not due to maternal effects (Rhen et al., 2011; Schroeder et al., 2016). In these species, sex determination is clearly a classic threshold trait determined by a combination of genes and the environment.

**Figure 4:**
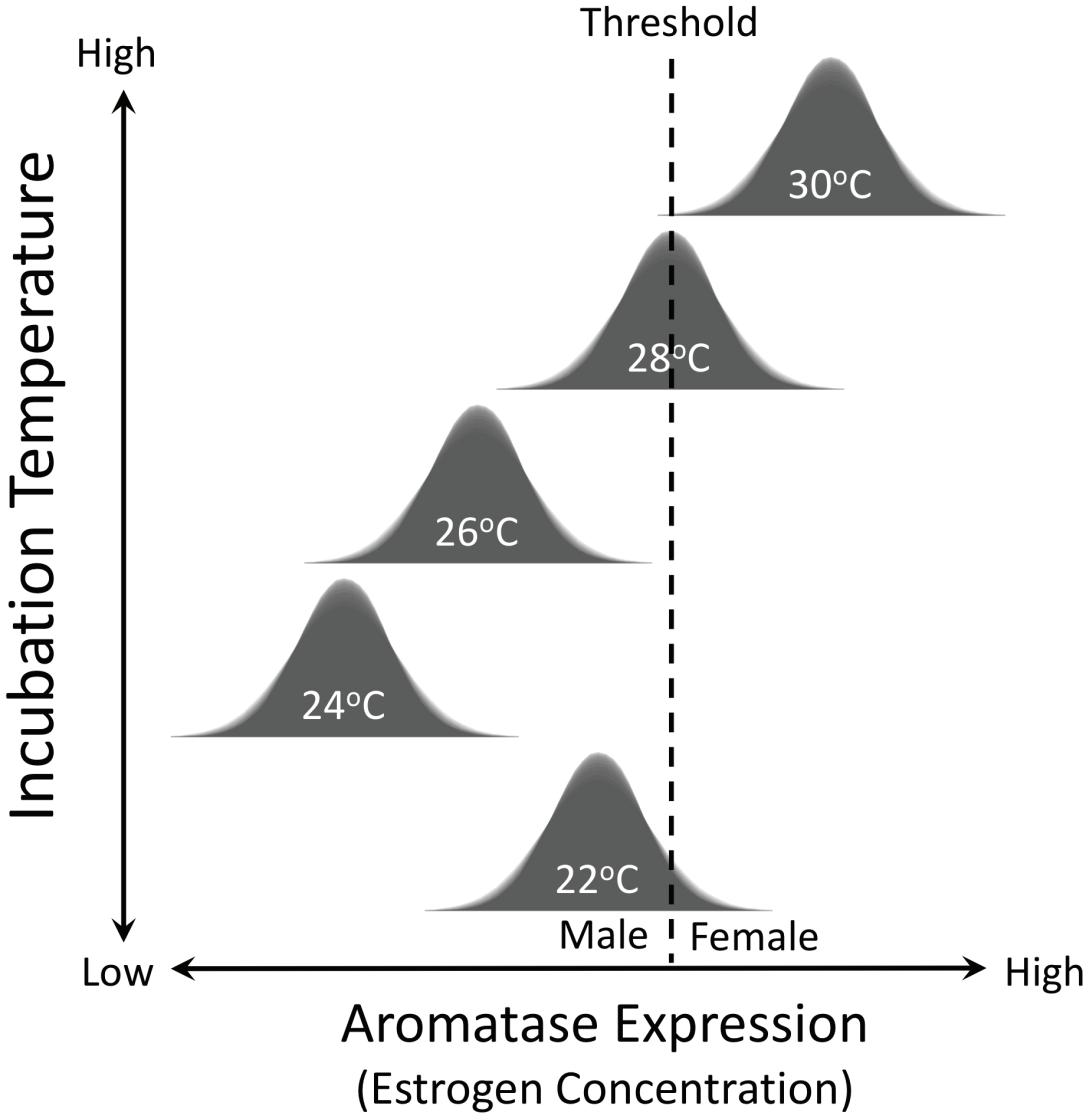
Threshold model for temperature-dependent sex determination. Incubation temperature during development influences expression of the aromatase enzyme, which synthesizes estrogens. Variation in expression of aromatase enzyme and the concentration of estrogen is normally distributed at each incubation temperature. If estrogen concentration in embryonic gonads is below a critical threshold, gonads develop into testes. If they are above this threshold, gonads develop into ovaries.

Temperature has also been shown to have quantitative effects on sex determination in TSD species. In other words, the magnitude and duration of exposure to male (or female) producing temperatures during the critical sex-determining period influences the likelihood of embryos developing testes or ovaries (Yntema, 1979; Mrosovsky and Pieau, 1991; Wibbels et al., 1991; Lang and Andrews, 1994; Merchant-Larios et al., 2010; Rhen et al., 2015). For example, 31°C has a more potent feminizing effect on snapping turtle embryos than does 29.5°C, which is closer to the pivotal temperature of 28.2°C in the focal population from Minnesota (Figure 3; Rhen et al., 2015). Conversely, 24°C is a more potent masculinizing temperature than 25°C, which in turn is more masculinizing than 26.5°C (Rhen et al., 2015). These observations imply that temperature is influencing production of some biochemical factor (or factors) in a quantitative way and that this signal is translated into a binary developmental response *via* a threshold-like mechanism (Figure 4). Aromatase, a steroidogenic enzyme that converts androgens into estrogens, is likely to be a key factor. If estrogen concentration in embryonic gonads is below a critical threshold, gonads develop into testes. If they are above this threshold, gonads develop into ovaries (Figure 4).

Indeed, ovary determination in TSD species is estrogen dependent in virtually all turtle, lizard, and crocodilian species that have been examined to date. Applying estrogens to embryos at male temperatures drives ovary formation, while aromatase inhibitors, which block estrogen synthesis, obstruct ovary development and induce testis development at temperatures that normally produce females or mixed sex ratios (Crews et al., 1994; Dorizzi et al., 1994; Rhen and Lang, 1994; Pieau et al., 1999; Pieau and Dorizzi, 2004; Freedberg et al., 2006; Lance, 2009; Rhen and Schroeder, 2010). In accord with these experimental manipulations, aromatase expression at female temperatures is higher than at male temperatures in several reptile species with TSD (Desvages and Pieau, 1992; Desvages et al., 1993; Lance, 2009; Rhen and Schroeder, 2010).

**Figure 5:**
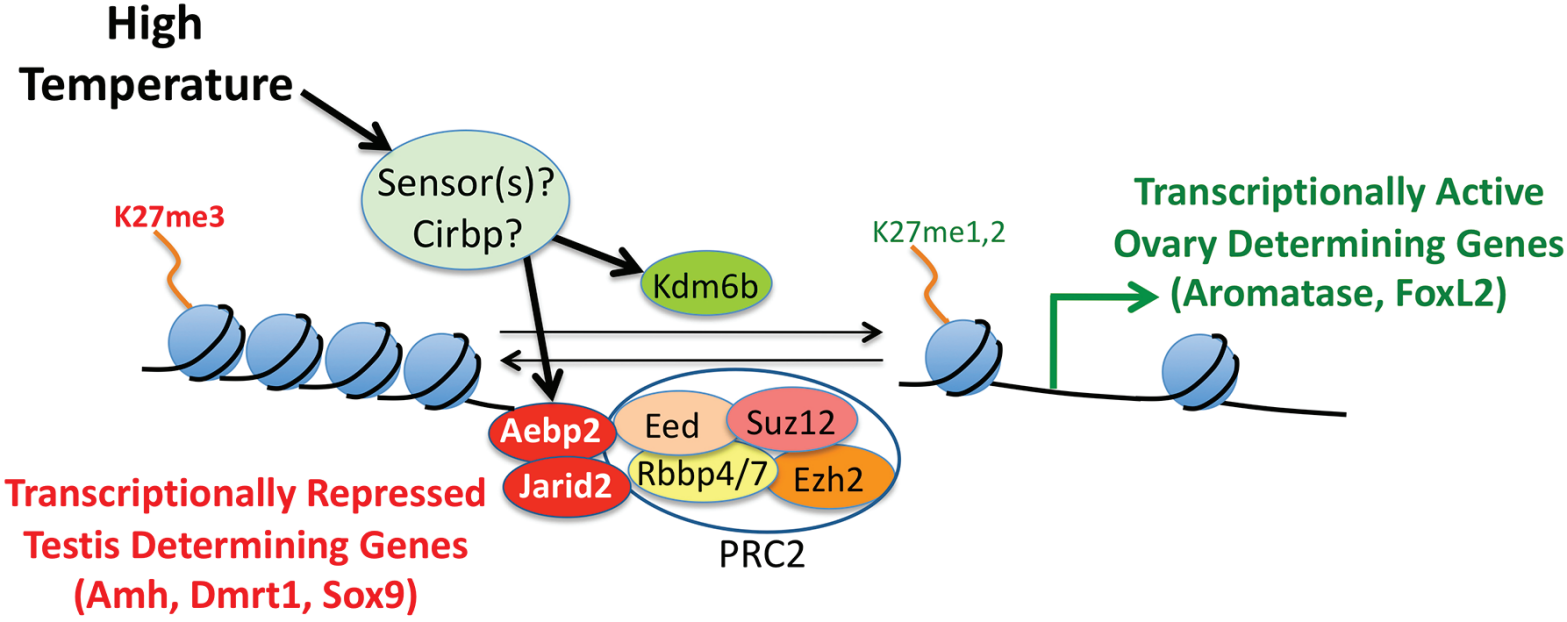
A conceptual model of the molecular mechanisms underlying temperature-dependent sex determination. Hypothetical temperature sensors influence expression and/or splicing of epigenetic regulators like Jarid2, Aebp2, and Kdm6b. In turn, these proteins influence methylation levels of histone H3 on lysine 27. Tri-methylation of H3K27 results in silencing of target genes, while mono- or di-methylation of H3K27 results in activation of target genes.

The specific mechanisms that regulate aromatase expression in embryonic gonads are unknown. We hypothesize that one or more genes encode sensors that are activated when temperature is in a particular range (Figure 5). The sensor (or sensors) then triggers the bipotential gonads to develop as ovaries. Recent studies suggest that epigenetic regulators of histone H3 methylation on lysine 27 (H3K27) may be involved in mediating temperature effects on expression of various sex-determining genes (Figure 5). In other vertebrates, Jumonji and AT-Rich Interaction Domain Containing 2 (Jarid2) helps recruit Polycomb Repressive Complex 2 (PRC2) to specific genomic locations (Healy et al., 2019). In turn, PRC2 methylates H3K27, which results in transcriptional silencing of target genes (Figure 5). In contrast, Lysine Demethylase 6B (Kdm6b) removes methyl groups from H3K27, thereby relieving repression (Figure 5).

In a study of bearded dragons (*Pogona vitticeps*), Deveson et al. (2017) found that Jarid2 and Kdm6b retain introns in temperature-induced ZZ females unlike ZZ males or ZW females. Similarly, differentially retained introns were detected at male- and female-producing temperatures in *Alligator mississippiensis* and *Trachemys scripta*, which are known to exhibit TSD. In yet another study, expression of Kdm6b (Ge et al., 2018) regulates Doublesex and Mab-3 Related Transcription Factor 1 (Dmrt1) expression. In turn, Dmrt1 acts as a transcription factor that is important for testis determination at male-producing temperatures in *Trachemys scripta*.

There is strong evidence that Cold-inducible RNA Binding Protein (Cirbp) plays a role in TSD in the snapping turtle (Schroeder et al., 2016). This gene displays temperature-dependent, allele specific expression. One allele is induced by exposure to a female producing temperature, while expression of the other allele is not affected by this thermal treatment. There is a strong genetic association between Cirbp genotype and the sex of hatchlings. When exposed to a female temperature for just 2.5 days, homozygotes for the temperature sensitive allele are four times more likely to develop ovaries than are heterozygotes, which are in turn four times more likely to develop ovaries than homozygotes for the temperature in-sensitive allele. This association extends to the family and population level and provides another mechanistic link between genes and the environment.

Despite evidence that aromatase, Jarid2, Kdm6b, and Cirbp are factors involved in mediating temperature effects on sex determination, the actual temperature sensors have yet to be identified in TSD species (Georges and Holleley, 2018). It will also be important to carry out mechanistic studies to understand how these genes interact with each other to determine sex.

## Temperature Effects on Quantitative Traits

While sex ratios provide a clear, albeit course, measure of temperature effects on cell, tissue, and organ differentiation, there are many other examples of persistent thermal effects on quantitative phenotypes in reptiles. Others have reviewed temperature effects on a wide range of traits (Rhen and Lang, 2004; Booth, 2018; Mitchell et al., 2018; Noble et al., 2018; While et al., 2018). Thus, we discuss just a few interesting examples of long-lasting effects on physiology and behavior.

### Incubation Temperature May Program Metabolism

Incubation has been shown to influence thyroid hormone physiology and metabolic rate. Resting metabolic rate and circulating thyroid hormone concentration decreased with increasing incubation temperature in hatchling snapping turtles that had been incubated across a wide range of temperatures (O’Steen and Janzen, 1999). Measurements were conducted in hatchlings held at the same ambient temperatures, which indicates that prior incubation temperature had a persistent effect on metabolic physiology. In another experiment in the same paper, eggs were incubated at two temperatures, one that produces males and one that produces females, and treated with triiodothyronine. Thyroid hormone treatment during embryogenesis had no effect on sex determination but increased developmental rate (treated turtles hatched sooner than controls), decreased hatchling mass, and raised the resting metabolic rate of hatchlings. Given that snapping turtles were studied a few days after hatching, longer-term studies will be required to distinguish whether these effects are simply due to thermal acclimation or are the result of developmental programming of metabolic phenotype. Nevertheless, studies of American alligators do suggest that disruption of thyroid hormone signaling during embryogenesis can program functioning of the hypothalamic-pituitary-thyroid axis as long as 10 months after hatching (Boggs et al., 2013).

### Incubation Temperature Programs Endocrine Phenotype

Incubation temperature influences steroid hormone synthesis during embryonic development. Exposure to different hormonal environments may in turn program physiological and behavioral traits. For instance, incubation temperature during embryonic development has been shown to affect circulating levels of estradiol-17β, progesterone and testosterone in juvenile red-eared slider turtles (Rhen et al., 1999). The circulating concentration of estradiol-17β, progesterone, testosterone, and dihydrotestosterone also differs among juvenile leopard geckos that were incubated at different temperatures (Rhen et al., 2005). Importantly, these effects on steroid levels persist into adulthood in the leopard gecko (Crews et al., 1998; Rhen et al., 2000). This strongly suggests that temperature during embryonic development directly or indirectly (*via* its effect on steroidogenesis) programs the hypothalamic-pituitary-gonad axis (Rhen and Crews, 2002). In accord with this inference, incubation temperature alters patterns of metabolic capacity in specific hypothalamic nuclei of leopard geckos in adulthood (Sakata et al., 2000; Sakata and Crews, 2003, 2004).

### Incubation Temperature Programs Growth Physiology

Incubation temperature has been shown to influence both embryonic and post-hatching growth rate in a number of reptiles (Noble et al., 2018; While et al., 2018). Thermal effects on embryonic growth rate can be attributed to a direct Q_10_ effect, whereas persistent effects on hatchlings and juveniles show thermal acclimation or permanent developmental effects on growth physiology. Cooler incubation temperatures produced hatchlings that grow significantly faster than hatchlings from the hottest incubation temperatures in the green anole (Goodman, 2008), the broad-snouted caiman (Pina et al., 2007), and the Murray River turtle (Micheli-Campbell et al., 2011). Incubation temperature has also been shown to influence post-hatching growth rate in numerous studies of the snapping turtle (Bobyn and Brooks, 1994; Rhen and Lang, 1995, 1999; O’Steen, 1998).

Rhen and Lang (1995) used experimental manipulations to decouple temperature and sex effects, revealing that incubation temperature programmed growth physiology independently of its effect on sex determination. In snapping turtles, temperatures that normally yield males produce faster growing turtles than temperatures that produce females. These studies examined growth in common gardens over several months to a year after hatching. Thermal acclimation normally occurs over a period of days or weeks so these incubation temperature effects on growth physiology are likely to be permanent. Indeed, incubation temperature affects adult body size in leopard geckos (Tousignant and Crews, 1995). Again, it is likely that temperature is altering neuroendocrine phenotype (i.e., the hypothalamus, pituitary, and growth hormone signaling). However, incubation temperature effects on subsequent growth do not always translate into differences in adult body size: incubation temperature has a temporary effect on body size in juvenile Australian jacky dragons (*Amphibolurus muricatus*) (Warner and Shine, 2005).

### Incubation Temperature Programs the Brain and Behavior

Several of the physiological traits discussed above are regulated by neuroendocrine mechanisms, so it is not surprising that temperature has persistent effects on the behavior of reptiles. An assortment of behaviors has been studied, including thermoregulation, righting and swimming performance, as well as sociosexual behaviors. Most of these studies have examined behavior in hatchlings or juveniles, but a few have shown embryonic programming of adult behaviors.

Thermoregulatory behavior of snapping turtles, for instance, is programmed by incubation temperature (O’Steen, 1998; Rhen and Lang, 1999). Both studies report a negative correlation between incubation temperature and the thermal environment selected by juvenile turtles. O’Steen (1998) measured mean preferred temperatures ranging from 24.5 to 28°C. In other studies of snapping turtles, mean temperatures selected in aquatic thermal gradients were 28 and 30°C for hatchlings (Williamson et al., 1989; Knight et al., 1990) and 28°C for adults (Schuett and Gatten, 1980). Selection of particular ambient temperatures is likely to influence growth rate because body temperature has a profound effect on growth. Williamson et al. (1989) found that hatchlings held at 15°C did not grow at all, while those held at 25°C grew seven-fold in mass over 1 year. Rhen and Lang (1999) similarly reported that snapping turtles held at 19°C grew very slowly or not at all while those held at 28°C grew rapidly. Turtles that had been incubated at cool and intermediate temperatures (24 and 26.5°C) were able to grow at 19°C, but turtles from a warm incubation temperature (29°C) did not grow at 19°C. These observations suggest incubation temperature programs the critical thermal minimum for growth. Further studies will be required to determine whether critical thermal maxima also differ and if preferred body temperatures match optimal temperatures for growth in snapping turtles from different incubation temperatures.

Micheli-Campbell et al. (2011) report that swimming behavior and performance in the turtle *Elusor macrurus* are altered by incubation temperature. Hatchling turtles from cool and intermediate temperatures swim more and have a stronger mean stroke force than turtles from the highest incubation temperature. Turtles from cool and intermediate temperatures are also able to right themselves more quickly when turned on their back. In contrast, yearling turtles from a warm temperature righted themselves faster than turtles from a cooler temperature in two other species *Graptemys ouachitensis* and *Trachemys scripta* (Freedberg et al., 2004). Extreme embryonic temperatures increased righting time relative to intermediate temperatures in *Caretta caretta* (Fisher et al., 2014). The mechanism underlying these differences in locomotor performance is unknown. However, these effects can last as long as 1 year.

**Figure 6:**
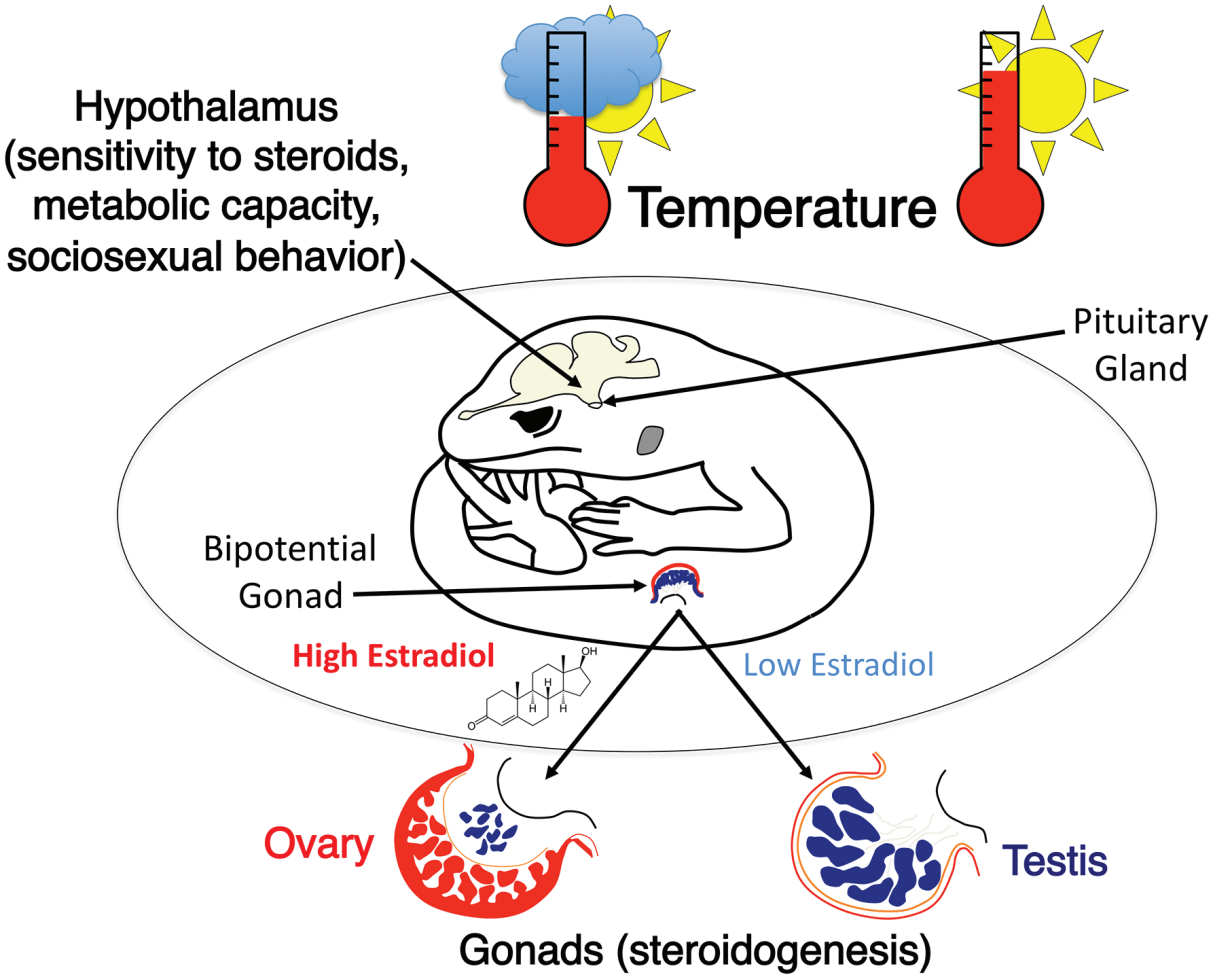
A graphical illustration of temperature effects on neural and gonad development in the leopard gecko. Incubation temperature determines sex and causes intrasexual variation in sociosexual behaviors. These differences in behavior are correlated with significant differences in metabolic capacity within specific hypothalamic nuclei as well as differences in sensitivity to sex steroids that activate sociosexual behaviors.

The impact of incubation temperature on behavior in adult leopard geckos of both sexes has been studied extensively (reviewed in Crews et al., 1998; Rhen and Crews, 2002; Sakata and Crews, 2004). Both embryonic temperature and gonadal sex affect sexual and aggressive behavior, sex steroid levels in the circulation, and the metabolic activity of several brain regions, including hypothalamic nuclei critical for the display of sociosexual behaviors (Figure 6). Sexual dimorphism in sociosexual behavior in leopard geckos follows the basic pattern found in mammals (Rhen and Crews, 1999, 2000). Females do not display male-typical scent marking or sexual behavior when treated with androgens that activate these behaviors in males. This indicates that sexual dimorphism in hormone responsiveness in adulthood is programmed at some point during ontogeny, presumably by sex differences in secretion of steroid hormones (Rhen et al., 2005).

In addition to pronounced sex differences, there is substantial intrasexual variation in sexual and aggressive behavior that is programmed by embryonic temperature. Male leopard geckos from a temperature that produces a female-biased sex ratio are more sexually active and less aggressive towards females than are males from a temperature that produces a male-biased sex ratio (Flores et al., 1994). Yet, temperature also affects endocrine physiology in adult males: males from a female-biased temperature have a lower ratio of androgens to estrogens when compared to males from a male-biased temperature (Tousignant and Crews, 1995; Crews et al., 1996).

To test whether the persistent effects of incubation temperature on behavior in adults were simply due to differences in hormone profiles in adulthood or to programming during early development, male leopard geckos from different temperatures were castrated and treated with the same levels of testosterone or dihydrotestosterone in adulthood. Males from the male-biased and the female-biased incubation temperatures behaved very differently even though they had the same levels of circulating hormones: males from the male-biased temperature scent marked more than males from the female-biased temperature (Rhen and Crews, 1999). Conversely, males from the female-biased incubation temperature were more sexually active (i.e., courted and mounted females more) than males from the male-biased temperature. Taken together, these findings demonstrate that embryonic temperature programs the male leopard gecko brain to respond differently to sex steroids in adulthood (i.e., classic organizational effects).

Incubation temperature effects on behavior in leopard geckos are correlated with physiological differences in the brain. More sexually active males from the female-biased temperature have greater metabolic capacity (i.e., cytochrome oxidase) in several brain regions than less sexually active males from the male-biased temperature. Incubation temperature effects on metabolic capacity in the preoptic area of the hypothalamus make sense because this region of the brain is essential for the display of male sexual behavior in all vertebrates, including leopard geckos (Edwards et al., 2004). Developmental temperature also influences metabolic capacity in brain nuclei that regulate agonistic behavior (Crews et al., 1996; Coomber et al., 1997). In summary, embryonic temperature clearly programs endocrine physiology, neurological phenotype, and behavior and may program metabolic physiology and some aspects of locomotor performance in a various reptilian species.

## Current Research on Epigenetic Mechanisms

The specific molecular factors that transduce temperature into a biological signal are largely unknown. However, there is increasing evidence that these hypothetical factors target epigenetic mechanisms, which provide a link to cell, tissue, and organ differentiation. Epigenetic mechanisms have been shown to play a role in environmental regulation of several traits in animals. In recent years, attempts have been made to survey epigenetic mechanisms to understand how temperature programs phenotype including studies of DNA methylation and histone modifications in several reptiles with TSD (Navarro-Martin et al., 2011; Matsumoto et al., 2013, 2016; Czerwinski et al., 2016; Yatsu et al., 2016; Fan et al., 2017; Radhakrishnan et al., 2017, 2018; Ge et al., 2018; Martín-del-Campo et al., 2019).

Whether genetically or environmentally programmed, developmental events like cell fate determination and cellular differentiation involve permanent changes in gene expression (Barsi et al., 2014). Post-translational modifications of histones and DNA methylation are key epigenetic marks that play a part in development by regulating cell-type and tissue-specific patterns of gene expression (Cedar and Bergman, 2009; Greer and Shi, 2012). Cytosine methylation (i.e., 5-methylcytosine) in particular is associated with gene silencing and is stably passed from mother to daughter cells during mitosis. In mammals, DNA methylation has context dependent effects on gene expression, depending on whether 5-methylcytosines are found at transcription start sites, in gene bodies, in repetitive DNA, or in enhancers and silencers (Jones, 2012).

Changes in DNA methylation mediate various environmental effects on phenotype. Temperature affects DNA methylation at the genome-wide level in many animals (Anastasiadi et al., 2017; Metzger and Schulte, 2017). A correlation between body temperature and overall DNA methylation level has been previously shown in vertebrates (Jabbari et al., 1997) and reptiles (Varriale and Bernardi, 2006). Differential DNA methylation at sex-determining genes may be a critical factor for sex determination in animals (Gorelick, 2003). In some TSD species, the promoter region of the aromatase gene shows tissue specific, sexually dimorphic DNA methylation, which is affected by temperature exposure during the sex-determining period. More specifically, higher methylation of particular cytosines is associated with lower aromatase expression in gonads of embryos at male-producing temperatures when compared to embryos at female-producing temperatures in two fish and one turtle species (Navarro-Martin et al., 2011; Matsumoto et al., 2013; Fan et al., 2017).

Differentially methylated regions have been reported in genome-wide studies of hatchling testes and ovaries in the painted turtle, *Chrysemys picta* (Radhakrishnan et al., 2017). Methylated DNA immunoprecipitation combined with next generation sequencing of the DNA (MeDIP-Seq) and analysis of CpG content in the genome shows sexually dimorphic DNA methylation at promoter, exon, intron, and inter-genic regions in the painted turtle genome. The study identified a total of 5,647 differentially methylated regions between male and female gonads including several genes reported to be involved in gonadal development. However, the observation of differential DNA methylation in hatchling gonads may reflect a role in maintenance of cell and organ fate rather than a role in sex determination in embryos incubated at male and female producing temperatures. Future studies should examine temperature effects on DNA methylation in embryos during the temperature sensitive period.

Radhakrishnan et al. (2018) tried to correlate temperature with epigenetic mechanisms by studying differences in expression of genes known to be involved in DNA methylation and histone modification along with some small RNAs in TSD (*Chrysemys picta*) and GSD (*Apalone spinifera*) turtle species. Several genes involved in DNA methylation (Dnmt3b) and histone methylation (Nsd1, Setd1a, Carm1, Prmt1, Ash1l, and Prdm2) were found to be differentially regulated between male and female producing temperatures in *Chrysemys picta* (Radhakrishnan et al., 2018). These results together underscore the importance of studying mechanisms involved in epigenetic regulation of gene expression and TSD.

Along with DNA methylation, histone modifications have been shown to play a crucial role in early embryonic development in reptiles (Feiner et al., 2018). Kdm6b is differentially expressed between gonadal transcriptomes from embryos incubated at male and female producing temperatures in TSD species *Alligator mississippiensis* (Yatsu et al., 2016) and *Trachemys scripta elegans* (Czerwinski et al., 2016). Ge et al. (2018) showed the sexually dimorphic expression of Kdm6b during the sex-determining period in *Trachemys scripta* embryos favors testis development. Another epigenetic regulator (Jarid2) shows temperature-dependent intron retention and differential expression of isoforms in male and female gonads in bearded dragons (Deveson et al., 2017). Jarid2 is currently being investigated as a potential TSD gene (Georges and Holleley, 2018).

## Temperature Effects on Development Plasticity in Light of Global Warming

In summary, incubation temperature during embryogenesis has significant effects on numerous hatchling phenotypes in reptiles. Many of the phenotypic differences examined are not simply due to direct temperature effects on rates of biochemical reactions (i.e., the Q_10_ effect). Neither are they the result of reversible physiological changes (i.e., thermal acclimation). Rather, they appear to be permanent developmental effects (i.e., fetal programming of phenotype). Indeed, some studies demonstrate temperature effects lasting into adulthood. Recent work suggests that epigenetic marks like DNA methylation and histone modifications may mediate the persistent effects of temperature in reptiles. In other organisms, such marks are involved in conferring cellular “memory” of environmental signals during early development (Bird, 2002; Ringrose and Paro, 2004; Hansen et al., 2008; Jones, 2012).

It is imperative to determine whether (1) these temperature effects translate to nature and (2) global warming is likely to have an impact on reptiles (and other ectotherms) in the wild. Studies of TSD species suggest the answer to both questions is yes. Variation in sex ratio is correlated with nest or water temperatures in nature in crocodilians, lizards, turtles, and fish (Janzen, 1994; Weisrock and Janzen, 1999; Wapstra et al., 2009; Simoncini et al., 2014; Pezaro et al., 2016; Jensen et al., 2018; Patricio et al., 2018; Honeycutt et al., 2019; Mitchell and Janzen, 2019; Tilley et al., 2019). These studies indicate that TSD is operative in nature. Moreover, warming temperatures over the last couple of decades are correlated with extremely female biased sex ratios in sea turtles from some rookeries, but not others (Hays et al., 2017). However, some laboratory studies that mimic fluctuating nest temperatures produce results that are not simply predicted by amount of time embryos spend above or below pivotal temperatures (Escobedo-Galván, 2013).

More research is clearly needed to assess the impact of temperature in natural nests on other traits in reptiles, but we expect that patterns observed at constant temperatures in laboratory studies will generally correlate with thermal effects in nature. If so, we can begin to predict the long-term impact of global warming. In the case of biased primary sex ratios in TSD species, there are several behavioral, physiological, and evolutionary changes that could mitigate the effects of climate change. Adult females may respond *via* changes in nesting phenology or selection of different thermal microenvironments for their nests (Hays et al., 2017; Jensen et al., 2018; Patricio et al., 2018). Genetic variation in thermosensitivity of embryos will also play a critical role in evolutionary responses. In snapping turtles, for instance, there is evidence of standing genetic variation for embryonic developmental rate (i.e., significant counter gradient variation) and for TSD (i.e., sex determination is heritable).

Research on the epigenetic mechanisms that mediate temperature effects in reptiles may more broadly inform our understanding of thermal biology. For example, PRC2 regulates histone methylation at the floral repressor gene and temperature-dependent vernalization in the model plant *Arabidopsis thaliana* (Coustham et al., 2012). Given that temperature has such a pervasive effect on living organisms, our hypothetical “temperature sensors” might also be highly conserved.

## Future Research Directions

While epigenetic mechanisms are likely to be important for understanding the pleiotropic effects of temperature on phenotype, we appear to be missing those specific factors that actually sense and transduce thermal variation into a biological signal. In other words, we are still in search of the physical link between the kinetic energy of molecules in living organisms and the specific molecular, cellular, and developmental mechanisms that produce distinct phenotypes. Therefore, one key area for future research is the identification of temperature sensors that induce specific phenotypes. One approach would be to work upstream from known mediators of thermal effects, like the aromatase gene in TSD species. Another approach would be to use random mutagenesis to identify loci that influence thermal reaction norms, but this would be limited to a handful of model organisms suitable for forward genetics. Once these hypothetical temperature sensors are found, an important set of questions will center on how temperature alters their molecular structure and function. Such studies will reveal how these sensors interact with and regulate epigenetic mechanisms.

A second vital area for research is the assessment of genetic variation for thermosensitivity and the identification of the specific DNA variants that cause this variation. Phenotypic and genotypic variation in thermosensitivity is necessary for evolutionary responses to a changing climate (Hoffmann and Sgrò, 2011). We only briefly discussed countergradient variation, but this pattern of genetic adaptation to thermal clines provides a good model for classical and molecular genetic analyses of thermosensitivity. Genetic variation in TSD is also a fertile area for study. The snapping turtle is an excellent model given the extent of variation in TSD both within and among populations. Ultimately, identification of temperature sensors and natural genetic variation in thermosensitivity will help us understand how life on Earth has adapted to thermal variation in the past and how it is likely to adapt to climate change now and in the future.

## Author Contributions

SS, DD, and TR prepared the draft and revised the manuscript.

### Conflict of Interest

The authors declare that the research was conducted in the absence of any commercial or financial relationships that could be construed as a potential conflict of interest.
